# Socioeconomic Differences in Walking Time of Children and Adolescents to Public Green Spaces in Urban Areas—Results of the German Environmental Survey (2014–2017)

**DOI:** 10.3390/ijerph18052326

**Published:** 2021-02-26

**Authors:** Julia Rehling, Christiane Bunge, Julia Waldhauer, André Conrad

**Affiliations:** 1 Department Environmental Hygiene, German Environment Agency, 14195 Berlin, Germany; christiane.bunge@uba.de (C.B.); andre.conrad@uba.de (A.C.); 2 Department of Epidemiology and Health Monitoring, Robert Koch Institute, 12101 Berlin, Germany; WaldhauerJ@rki.de

**Keywords:** environmental justice, public green spaces, health inequalities, environmental inequalities, socioeconomic position

## Abstract

Public green spaces have a high potential for a positive impact on people’s health and wellbeing, especially in urban areas. Studies on environmental justice indicate socially unequal access possibilities to urban green spaces. This article presents results on associations between individual socioeconomic position (SEP) and walking time from home to public green spaces in young people living in urban areas with more than 20,000 inhabitants in Germany. Data were derived from the German Environmental Survey for Children and Adolescents 2014–2017 (GerES V), the environmental module of the German Health Interview and Examination Survey for Children and Adolescents (KiGGS Wave 2). The sample comprises 1149 participants aged 3 to 17 years. A total of 51.5% of the participants reach a public green space on foot within five and 72.8% within ten minutes from home. The lower the participant’s SEP, the longer the walking time. Logistic regression models controlling for age group, sex, migration background, and region of residence show that participants with a low SEP have a significantly higher risk (odds ratio = 1.98; 95% confidence interval: 1.31–2.99) of needing more than ten minutes to walk from home to a public green space than participants with a high SEP. GerES V data indicate that young people living in urban areas in Germany do not equally benefit from the health-promoting potential of green spaces, which is an important aspect of environmental health inequalities.

## 1. Introduction

Almost half of the world’s children live in urban areas [1]. In Germany, 60% of families with children under the age of 18 lived in cities with 20,000 inhabitants or more in 2019 [2]. The acceleration of urban demographic growth, conflicts about the use of space, and the increasing impacts of climate change put especially urban areas under pressure. Addressing these challenges is one of the key tasks in order to provide healthy and sustainable living environments for the inhabitants of urban areas [3].

Urban green spaces—as part of the concept of environmental health [4]—play an important role in this context. They provide areas for physical activities, stress reduction, relaxation, and social interaction, and hence offer a high potential for beneficial effects on health and well-being, for both adults and children [5,6,7,8]. Empirical evidence on the effects of urban green spaces on children’s health shows that they are positively associated with physical activity [9,10], reduced obesity [11], intellectual and behavioural development [12] and a lower risk of a wide range of mental disorders later in life [13]. Some findings even suggest an enhanced functioning of the immune system through contact with green spaces [14,15]. Another study demonstrates improved social relationships in 10 to 17 year olds due to leisure activities in urban forests and public green spaces [16]. All of these aspects are essential for a healthy development, as health is not merely the absence of disease or infirmity, but a state of complete physical, mental and social well-being [17].

Additional beneficial health effects of urban green spaces are discussed in the context of ecological and climate regulation. Urban green spaces can substantially reduce noise and air pollution [5,18,19]. Moreover, they can have a cooling effect on the neighbouring development and contribute to a significant lowering of the urban heat island effect by providing shade, fresh air lanes and reducing the temperature on hot days [20,21]. With the development of an increasing number of hot days and tropical nights as a result of climate change, easily reachable urban green spaces become more important, especially for vulnerable population groups such as children; they provide opportunities to escape from the heat [5].

Most recently, the COVID-19 pandemic demonstrates the vital importance of urban green spaces once again. Particularly when there are restrictions on individual movement, social contact and public life in general, nearby urban green spaces can have a decisive balancing and health-promoting function. This has already been pointed out in different publications [22,23,24]. In an online survey carried out in various European countries within the first COVID-19 wave in 2020, respondents with access to outdoor spaces were less likely to report symptoms of depression and anxiety than individuals with no accessible outdoor spaces [25]. Based on two surveys in the UK and Scotland, Olsen and Mitchell [26] also reported mental health benefits from green and open spaces during lockdown. Further, they pointed out that the use of green space was polarised during lockdown: while some people did increase their frequency of use and time spent outside, many made fewer or no visits at all. The authors state that the lockdown did not reduce socioeconomic inequalities in the use of green spaces, but may have made them worse.

It is known that there are pronounced social inequalities with regard to access to health-promoting environmental resources and health-related environmental pollution [27,28,29]. Researchers discuss and investigate these findings under the term of environmental justice [30]. With respect to environmental resources, studies indicate that people with a low socioeconomic position (SEP) or entire deprived neighbourhoods often have poorer access to urban green spaces than people with a higher SEP and, respectively, less deprived neighbourhoods [31,32,33,34]. In Germany, the question of equal access to health-promoting urban green spaces has also become an important research topic on environmental justice in recent years [35]. Results show socioeconomic differences on the individual and the contextual level. Schüle et al. [36], for example, found out that a more deprived neighbourhood in the city of Munich is associated with a lower availability of green spaces. At the individual level, a low household income of families in the cities of Munich and Leipzig was related to a lower degree of vegetation in the neighbourhood [37]. Wüstemann et al. [32] detected a positive correlation between individual income, individual education and the number of accessible green spaces in the residential area for German major cities with more than 100,000 inhabitants. In a study by Shrestha et al. [38], the extent of environmental inequality in terms of distance to green spaces (including parks and forests) in the city of Dortmund was even larger compared to inequalities concerning environmental burdens such as air pollution and noise.

Apparently, the preconditions to benefit equally from the health-promoting potential of urban green spaces are not the same for all population groups due to different access possibilities in the neighbourhood. Research indicates that households with children generally have more urban green space in their close neighbourhood in comparison to childless households [32]. Although there is evidence on the socially unequal distribution of green spaces in urban areas in Germany, results on how children are affected by this situation are limited or only available for a certain region [39].

The aim of the following analysis is to examine data of the German Environmental Survey for Children and Adolescents 2014–2017 for associations between children’s SEP and the time they need to walk from home to health-promoting public green spaces in urban areas in Germany. It is the first nationwide population-representative study in urban areas in Germany that provides information on how long children and adolescents actually need on foot from home to public green spaces and how the subjective walking time differs between population subgroups. Investigating the accessibility of public green spaces as health-promoting areas according to socioeconomic position, we aim for a better understanding of environmental inequalities in young people living in urban areas in Germany and insight into the broader context of health inequalities among children and adolescents.

## 2. Materials and Methods

### 2.1. Data Base and Study Sample

The German Environmental Survey for Children and Adolescents 2014–2017 (GerES V) is a large-scale cross-sectional study carried out by the German Environment Agency [40]. It is the environmental module of the German Health Interview and Examination Survey for Children and Adolescents (KiGGS Wave 2) of the Robert Koch Institute [41]. KiGGS Wave 2 was approved by the Ethics Committee of the Hannover Medical School (No. 2275–2014) [41]. GerES V received approval of the Ethics Committee of the Berlin Chamber of Physicians (No. Eth-14/14).

GerES V is a nationwide representative study on children and adolescents living in Germany and provides information on 3 to 17-year-old children and adolescents and their exposure to health-relevant environmental pollutants, as well as information on environmental health resources in their living environment.

The sample comprises information on 2294 participants. The relationship between SEP and green space availability differs between urban and rural areas in Germany due to enormous structural differences [37]. For this reason, we focused on participants who live in urban areas in Germany with a population of 20,000 inhabitants and more. This accounts for 56% of the GerES V sample, which is comparable to the overall proportion in Germany [2]. To categorise the community size according to the number of inhabitants, the political community size class (as of 31 December 2015) of the current participant’s address was used.

### 2.2. Variables and Measurement

#### 2.2.1. Dependent Variable: Walking Time to a Public Green Space

In personal computer-assisted interviews, parents or legal guardians of the 3 to 17 year olds were asked how long their child needs to walk from home to certain locations presented in a list. One of the items on the list referred to the walking time to a park or a public green space, and another one to the walking time to a forest. We combined those two aspects, defining our dependent variable as the shortest reported time participants need to walk from home to either a park, a public green space or a forest—for reasons of simplicity in the following only referred to as “public green spaces”. The respondents could choose between five possible response categories: 1 to 5 min, 6 to 10 min, 11 to 20 min, 21 to 30 min or more than 30 min. Further, they could indicate that they do not know the answer to this question.

For the regression analysis, the variable on walking time was binarily coded to distinguish between those participants who need a reasonable amount of time to walk to public green spaces and those whose walking time appears unreasonable. The question on the maximum time people should need to walk from home to a public green space, in order to benefit from its health-promoting potential, is increasingly being discussed in science and practice. The brief for action on urban green spaces of the WHO regional office for Europe states “[a]s a rule of thumb, urban residents should be able to access public green spaces of at least 0.5–1 hectare within 300 metres’ linear distance (around 5 minutes’ walk) of their homes” [3]. The inclusion of a minimum size in the accessibility analysis is explained by the recognition that green spaces only allow an attractive and broad use when they exceed a certain size [42]. Other sources determine an easy walking distance simply as a ten minute walk [43]. In Germany, there are also currently no standard indicators, but some orientation values exist. A quick and direct reachability with an orientation value of a five minute walk approximately has emerged as a criterion on how urban green spaces should be designed [5]. The Federal Institute of Research on Building, Urban Affairs and Spatial Development recommends an accessibility to nearby urban green spaces (≥1 ha) at a distance of 300 m (≈500 m on foot) and an accessibility to larger urban green spaces (≥10 ha) at a distance of 700 m (≈1000 m on foot). A specification in minutes is missing.

A uniform benchmark for a reasonable walking time is hard to find. Further, GerES V does not provide data on the size of the green spaces in the neighbourhood. This is why we concentrated on the walking distance in minutes and decided to investigate two possible cut-off values. First, we analyse socioeconomic differences in the risk of needing more than five minutes, and second, of needing more than ten minutes to an urban green space.

#### 2.2.2. Independent Variable: Socioeconomic Position (SEP)

Children’s SEP is the main independent variable of interest. It is part of the KiGGS Wave 2 dataset. Operationalised as a household characteristic, it is a composite multidimensional index based on information on the parents’ education level, occupational status and disposable net household income [44]. A low SEP represents children and adolescents who grow up in the least socioeconomically affluent families (the lowest 20% of the German population, regarding the multidimensional index). A high SEP depicts those who grow up in the most affluent families (the highest 20% of the German population), and a medium SEP comprises all children and adolescents of a broadly defined middle. For further information on the SEP index construction, see [44].

#### 2.2.3. Other Model Variables: Sociodemographic Factors

Additional sociodemographic factors were included in the statistical model as control variables. First of all, the participant’s age group was considered. We assume that walking time decreases with increasing age group because adolescents usually walk faster than younger children. Furthermore, the participant’s sex was included to test whether there are any differences in reported walking time between female and male participants. Another factor taken into account was the region of residence, distinguishing between participants who live in an urban area with more than 20,000 inhabitants in the former East and West Germany (based on the region where the sampling took place). Due to different urban development programs before and after the German reunification, there could be differences in public green space provision in the former East and West Germany that might influence analysis results [45]. The participants’ migration background could also be related to the walking time from home to public green spaces. A reason for this might be discrimination in the housing market, impeding an equal chance to live in green neighbourhoods for participants with migration background [46]. Although SEP and migration background are significantly correlated in GerES V (Kendall-Tau-b: −0.27, *p* < 0.001), the migration background can represent further discrimination paths in this context [47]. Information on the migration background is based on the country of birth of the child and their parents as well as the parents’ nationality. The three-stage variable distinguishes between having no, a one-sided or a two-sided migration background. A one-sided migration background is assumed if one of the parents was not born in Germany or does not have German citizenship. A two-sided migration background is assumed when either both parents were not born in Germany, but the participant was, or the participant themselves immigrated from another country and at least one parent was not born in Germany [48].

### 2.3. Statistical Analyses and Weighting

To analyse associations between the two main variables of interest, information on walking time is observed for each socioeconomic group in a cross tabulation. Kendall-Tau-b is used to test for a significant relationship, its direction and its strength. To evaluate which socioeconomic group has the highest risk of needing longer than five, respectively, ten minutes on foot to a public green space, logistic regression models were calculated controlling for the sociodemographic variables mentioned above. Resulting odds ratios (OR) express the factor by which the risk of needing more than five, respectively, ten minutes is increased in the low and medium socioeconomic group in comparison to the high socioeconomic group, which is the reference category.

The sample counts are reported unweighted. Reported frequencies were conducted with a weighting factor. It considers the multi-stage survey design of KiGGS Wave 2 and is based on the population structure in terms of age, gender, place of residence and education at the time of the first KiGGS survey (as of 31 December 2004) [49]. Like this, reported percentages can be interpreted as representative frequencies for all children and adolescents aged 3 to 17 years who live in urban areas in Germany with more than 20,000 inhabitants.

Logistic regression models were performed unweighted in order to avoid biased estimations of the factors that are included both in the regression model and in the weighting factor. Nevertheless, we considered the correlation of the participants within a municipality (sample point). Therefore, the analysis was performed using the complex sample procedure in the IBM SPSS 25 statistic software [50].

## 3. Results

### 3.1. Sample Description

Table 1 presents the description of the study sample according to all considered variables. A total of 51.5% of the parents from urban areas in Germany with more than 20,000 inhabitants stated that their child can walk to a public green space within five minutes from home. A further 21.3% need six to ten minutes, which means that 72.8% of the children and adolescents reach a public green space on foot within ten minutes and 27.2% need more than ten minutes. Almost 10.0% of the participants need more than 20 min to walk from home to a public green space, and nearly 5.0% walk longer than 30 min.

### 3.2. Bivariate Analysis: Walking Time according to Children’s SEP

**Figure 1:**
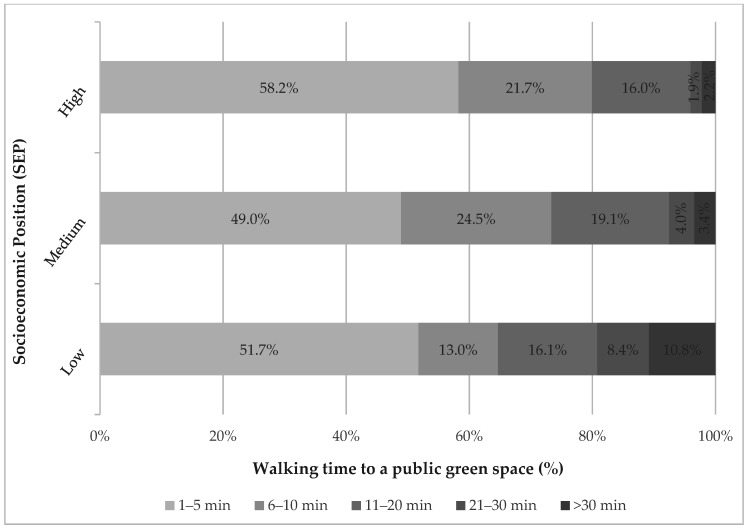
Walking time to a public green space from home by socioeconomic position (SEP) in children and adolescents living in urban areas in Germany (information provided by parents). N = 1110 (unweighted); % weighted according to data on the residential population of Germany; Kendall-Tau-b = −0.1 (*p* = 0.002).

How long children and adolescents walk from home to a public green space differs significantly by their SEP (*p* < 0.01) (Figure 1). In general, the lower the SEP, the longer the walking time. The value −0.1 (Kendall-Tau-b) indicates a weak negative association. Considering the five minute cut-off, 58.2% of children and adolescents with a high SEP reach a public green space within five minutes. In contrast, 48.9% of those with a medium SEP and 51.7% of those with a low SEP can reach a public green space within five minutes. Looking at the ten minute cut-off, socioeconomic differences are more pronounced and follow a social gradient: while 79.9% of children and adolescents with a high SEP need ten minutes at the most on foot from home to a public green space, 73.5% of those with a medium SEP and only 64.7% of those with a low SEP can reach a public green space within ten minutes.

The longer the walking time is, the more pronounced the socioeconomic differences become. A total of 8.4% of the participants with a low SEP walk between 21 and 30 min to a public green space. In comparison, 4.0% of those with a middle SEP and 1.9% of the participants with a high SEP walk between 21 and 30 min to a public green space. Only 2.2% of the participants with a high SEP need more than 30 min to walk to a public green space, while it is 3.4% of those with a medium SEP and 10.8% of those with a low SEP that walk more than 30 min to the next public green space.

### 3.3. Multivariate Analysis: Logistic Regression Model

Participants with a low and medium SEP have a higher risk of walking more than five, respectively, ten minutes to a public green space than children and adolescents with a high SEP, also after controlling for age group, sex, migration background and region of residence (Table 2). Looking at the five minute cut-off, coefficients show a social gradient, but are rather small and statistically not significant. Taking up the ten minute cut-off, results also indicate a social gradient and a statistically significant association for the low SEP group; the risk of walking more than ten minutes from home to a public green space is almost two times higher for participants with a low SEP compared to those with a high SEP. For participants with a medium SEP, the risk of walking longer than ten minutes to a public green space is 1.19 times higher compared to those with a high SEP.

The consideration of the other model variables reveals that age group is significantly associated with walking time. Three to five year olds have a 2.39 times (respectively, 3.15 times) higher risk of walking longer than five (respectively, ten) minutes to a public green space compared to 14 to 17 year olds. For the 6 to 10 year olds and the 11 to 13 year olds, there is also a higher risk of walking longer than five, respectively, ten minutes in comparison to the 14 to 17 year olds. All other model variables except age show no significant associations with the walking time examined.

## 4. Discussion

Our analysis shows that 51.5% of the children and adolescents living in urban areas in Germany with more than 20,000 inhabitants walk up to five minutes from home to a public green space. A quick and direct accessibility of public green spaces within the meaning of the orientation value of five minutes often recommended for urban areas in the corresponding literature is not given for almost half of the participants. A further 21.3% reach a public green space on foot within ten minutes. This means that almost one third of the children and adolescents who live in urban areas cannot walk to a public green space within ten minutes from home.

Furthermore, there are socioeconomic differences in the walking time to public green spaces: the lower the SEP, the longer the walking time from home to an urban public green space. This association becomes particularly visible with increasing walking time. Logistic regression models generally confirm previous bivariate findings: Using the five minute cut-off as a reasonable walking time to an urban public green space, the model shows that participants with a low and a middle SEP have a higher risk of needing more than five minutes from home to a public green space compared to those with a high SEP. Results were statistically not significant at this point. Taking the ten minute cut-off as an acceptable walking time displays a pronounced association in the form of a social gradient with statistically significant differences between low and high SEP participants. Additionally, our analysis revealed that especially younger age groups have a significantly higher risk of needing more time to walk from home to a public green space in comparison to the 14 to 17-year-old participants, supporting the fact that age is a relevant factor for walking time too. Although not focused on in the analysis, this finding shows that proximity and a quick and easy reachability of urban public green spaces from home are also of particular importance for small children. Socioeconomic and physical conditions might overlap at this point, highlighting the need for a more detailed analysis of the interaction between socioeconomic and sociodemographic factors in future research.

Our findings are generally comparable to other study results on socioeconomic differences in green space accessibility in urban areas, both internationally [31,33,34] and in Germany [32,36,37]. However, a detailed comparison of our analysis results with the literature is limited because how inequalities in environmental health resources are examined empirically varies greatly [35]. First of all, there are major differences in what is understood by a public green space [51]. Depending on research discipline and focus, definitions can include, for example, parks, forests, trees, allotments, playgrounds and cemeteries. Additionally, researchers sometimes analyse green spaces in combination with blue spaces [31]—another health-relevant aspect of outdoor environments that prominently features water and is accessible to humans [52]. GerES V data also provide information on the walking time from home to blue spaces, more precisely to a beach, lake, stream or river. Nevertheless, we take this topic in urban areas and its health-promoting effects as a separate one and as strongly dependent on the season. Therefore, we did not consider blue spaces in our analysis. Future GerES V data evaluation could take up on that topic in the context of environmental justice in order to provide deeper insight into relevant health associations. Another reason that limits the comparison of study results is the great heterogeneity on how access to public green spaces is operationalised [36]. Many studies use objective measures of distance, quality and quantity through geographic information systems (GIS) [34]. GerES V data only provide subjective information on children’s and adolescents’ walking time to public green spaces, which is a limitation. Additionally, details on the public green spaces’ quality, quantity and time of use have not been recorded. An analysis of participants’ housing coordinates in combination with objective GIS data would be a useful extension in order to validate and expand our findings based on subjective data. Similarly, it could be beneficial to collect additional information on the parents’ walking time as a comparative measure to their evaluation of the children’s walking time. However, the self-reported evaluation of walking times is also of value and it should be recognised that objective measures do not necessarily reflect subjective ones [31]. Schüle et al. summarise that some studies, for example, found that subjectively perceived green space availability is more strongly related to the actual individual use of green space and walking behaviours than objectively measured availability [31]. Further research in this area is needed.

Looking beyond GerES V, the German Environment Agency—in cooperation with the Robert Koch Institute—intends to carry out the 6th German Environmental Survey (GerES VI). The study will evaluate the health-relevant environmental burden and resources of the adult population in Germany. It is planned to expand the former question on walking time to public green spaces by subjective information on the frequency of use, quality of public green spaces and type of activities which are performed there. This is a decisive expansion allowing a more detailed analysis with respect to inequalities in environmental health resources in Germany.

Another limitation of the present study is that we did not consider the topic of private green spaces in urban areas, such as gardens. In GerES V, for almost 90% of the children and adolescents access to a garden or a green backyard has been reported. However, this separate questionnaire item could not be included in our analysis, as it does not indicate whether those gardens and yards are private or how far they are away from home. Research demonstrates that proximity and good accessibility are particularly important for daily short-term recreation. Access possibilities to urban green spaces thus can have an impact on the frequency and duration of stay [37]. In this context, Krekel et al. [53] showed that in addition to the size, it is even more proximity to urban green spaces that is significantly positively associated with life satisfaction. Furthermore, public and private green spaces have unique functions and meanings to people, showing that they are not just simple substitutes for each other [54]. Yet, an additional investigation of the role of private green spaces and living conditions might be beneficial for the understanding of social inequalities in environmental health resources.

The leading assumption behind our analysis is that children and adolescents who need more time to walk to urban public green spaces have a lower chance to benefit from their health-promoting potential. Our study underlines the hypothesis that less favoured social groups are more likely to encounter unfavourable health conditions and are less able to profit from health-promoting offers. To what extent the detected social disparities in walking time to public green spaces explain social inequalities in health was not the subject of the investigation. This topic should be focused on in future research.

The strength of our analysis is that it is the first one presenting population representative data on the time children and adolescents living in urban areas in Germany with more than 20,000 inhabitants need to walk to a public green space. At the same time, it provides representative information on socioeconomic differences in children and adolescents. This emphasises the importance of conducting a collaborative study and using a joint sample of GerES V with data of the KiGGS Wave 2 study, allowing a combined analysis of environmental health resources together with socioeconomic and sociodemographic factors. Poor access to public green spaces does not explain the whole range of health inequalities at a young age, and yet it is important to see the overall relationship between unequal chances for life and health and to understand them as part of the whole.

## 5. Conclusions

GerES V data reveal that children and adolescents with a low or medium SEP living in urban areas in Germany need more time to reach a public green space in their neighbourhood than children and adolescents with a high SEP. Thus, it can be inferred that children and adolescents with a low or medium SEP in urban areas in Germany have poorer access to public green spaces close to their homes than better-offs. In view of the well-known health benefits of public green spaces, this unequal access could increase existing health inequalities among children and adolescents in Germany. Besides socioeconomic differences, our results indicate the relevance of young age for a prolonged walking time to public green spaces. This shows that it is important to consider socioeconomic as well as sociodemographic aspects of residents when designing healthy living environments in order to provide access opportunities for a diverse range of users [13].

In our analysis, the walking time is exclusively based on subjective parental reports. A next step in further elucidating this topic would be to also take objective data into account. In this context, spatial data on public green spaces promise to be of particular benefit.

Although socioeconomic and sociodemographic differences in access to public green spaces may only explain parts of the large social inequalities in health, they underline the fact that there are many facets of disadvantage in wide areas of everyday life. These disadvantages accumulate in total and over the life course. Identifying and addressing these multiple burdens in a targeted manner, especially among the most vulnerable, must be the focus of future research and policy-making.

## Figures and Tables

**Table 1:** Description of the study sample [% (N)] ^1^.

**Walking time to a public green space**	
1–5 min	51.5 (604)
6–10 min	21.3 (245)
11–20 min	17.7 (185)
21–30 min	4.6 (47)
>30 min	4.9 (52)
Missing	(16)
**Socioeconomic Position**	
Low	23.5 (132)
Medium	55.0 (627)
High	21.5 (367)
Missing	(23)
**Age group**	
3–5 years	20.0 (214)
6–10 years	34.0 (372)
11–13 years	18.6 (272)
14–17 years	27.3 (291)
Missing	(0)
**Sex**	
Female	49.7 (599)
Male	50.3 (550)
Missing	(0)
**Migration background**	
One-sided	12.9 (138)
Two-sided	24.9 (173)
No migration background	62.2 (818)
Missing	(20)
**Region of residence**	
Former East Germany (incl. East Berlin)	13.3 (297)
Former West Germany (incl. West Berlin)	86.7 (852)
Missing	(0)

^1^ N = 1149 (unweighted); % weighted according to data on the residential population of Germany.

**Table 2:** Odds Ratios based on logistic regression analysis of the relationship between walking time to a public green space and socioeconomic position, regarding age group, sex, migration background and region of residence.

	Walking Time to a Public Green Space	
	>5 min	>10 min
**Socioeconomic Position**		
Low	1.20 [0.82–1.75]	1.98 [1.31–2.99] **
Medium	1.06 [0.82–1.36]	1.19 [0.86–1.64]
High (Ref.)	---	---
**Age group**		
3–5 years	2.39 [1.55–3.70] ***	3.15 [2.04–4.86] ***
6–10 years	1.14 [0.82–1.57]	1.42 [0.98–2.05]
11–13 years	1.20 [0.82–1.75]	1.47 [0.91–2.39]
14–17 years (Ref.)	---	---
**Sex**		
Female	1.15 [0.93–1.43]	1.10 [0.85–1.40]
Male (Ref.)	---	---
**Migration background**		
One-sided	1.23 [0.82–1.85]	1.00 [0.66–1.52]
Two-sided	1.28 [0.87–1.87]	1.15 [0.73–1.82]
No migration background (Ref.)	---	---
**Region of residence**		
Former East Germany (incl. East Berlin)	0.84 [0.63–1.13]	0.74 [0.51–1.08]
Former West Germany (incl. West Berlin) (Ref.)	---	---

** (*p* < 0.01), *** (*p* < 0.001).

## Data Availability

Data can be made available upon request (Scientific Use File).

## References

[1] United Nations Children’s Fund (UNICEF) (2012). Children in an Increasingly Urban World.

[2] Destatis (2020). Bevölkerung und Erwerbstätigkeit. Haushalte und Familien. Ergebnisse des Mikrozensus.

[3] World Health Organization (WHO) (2017). Urban Green Spaces: A Brief for Action.

[4] Harper S., Wexler P. (2014). Environmental Health. Encyclopedia of Toxicology.

[5] Claßen T. (2018). Urbane Grün- und Freiräume—Ressourcen einer gesundheitsförderlichen Stadtentwicklung. Planung für Gesundheitsfördernde Städte.

[6] Intelmann D. (2019). Healthy City-Einführung in ein Stadtkonzept.

[7] World Health Organization (WHO) (2016). Urban Green Spaces and Health. A Review of Evidence.

[8] Kabisch N., van den Bosch M., Lafortezza R. (2017). The health benefits of nature-based solutions to urbanization challenges for children and the elderly—A systematic review. Environ. Res..

[9] Almanza E., Jerrett M., Dunton G., Seto E., Pentz M.A. (2012). A study of community design, greenness and physical activity in children using satellite, GPS and accelerometer data. Health Place.

[10] Ward J.S., Duncan J.S., Jarden A., Stewart T. (2016). The impact of children’s exposure to greenspace on physical activity, cognitive development, emotional wellbeing, and ability to appraise risk. Health Place.

[11] Wolch J., Jerrett M., Reynolds K., McConnell R., Chang R., Dahmann N., Brady K., Gilliland F., Berhane K. (2011). Childhood obesity and proximity to urban parks and recreational resources: A longitudinal cohort study. Health Place.

[12] Bijnens E.M., Derom C., Thiery E., Weyers S., Nawrot T.S. (2020). Residential green space and child intelligence and behavior across urban, suburban, and rural areas in Belgium: A longitudinal birth cohort study of twins. PLoS Med..

[13] Engemann K., Pedersen C.B., Arge L., Tsirogiannis C., Mortensen P.B., Svenning J.-C. (2019). Residential green space in childhood is associated with lower risk of psychiatric disorders from adolescence into adulthood. Proc. Natl. Acad. Sci. USA.

[14] Kuo M. (2015). How might contact with nature promote human health? Promising mechanisms and a possible central pathway. Front. Psychol..

[15] Braubach M., Egorov A., Mudu P., Wolf T., Ward Thompson C., Martuzzi M., Kabisch N., Korn H., Stadler J., Bonn A. (2017). Effects of Urban Green Space on Environmental Health, Equity and Resilience. Nature-Based Solutions to Climate Change Adaptation in Urban Areas: Linkages between Science, Policy and Practice.

[16] Seeland K., Dübendorfer S., Hansmann R. (2009). Making friends in Zurich’s urban forests and parks: The role of public green space for social inclusion of youths from different cultures. Forest Policy Econ..

[17] World Health Organization (WHO) (2020). Basic Documents: Forty-Ninth Edition (Including Amendments Adopted Up to 31 May 2019).

[18] Klingberg J., Broberg M., Strandberg B., Thorsson P., Pleijel H. (2017). Influence of urban vegetation on air pollution and noise exposure—A case study in Gothenburg, Sweden. Sci. Total Environ..

[19] Baró F., Gómez-Baggethun E., Kabisch N., Korn H., Stadler J., Bonn A. (2017). Assessing the Potential of Regulating Ecosystem Services as Nature-Based Solutions in Urban Areas. Nature-Based Solutions to Climate Change Adaptation in Urban Areas. Theory and Practice of Urban Sustainability Transitions.

[20] Kabisch N., van den Bosch M.A., Kabisch N., Korn H., Stadler J., Bonn A. (2017). Urban Green Spaces and the Potential for Health Improvement and Environmental Justice in a Changing Climate. Nature-Based Solutions to Climate Change Adaptation in Urban Areas: Linkages between Science, Policy and Practice.

[21] Bowler D.E., Buyung-Ali L., Knight T.M., Pullin A.S. (2010). Urban greening to cool towns and cities: A systematic review of the empirical evidence. Landsc. Urban Plan..

[22] Gillis K. (2020). Nature-based restorative environments are needed now more than ever. Cities Health.

[23] Kleinschroth F., Kowarik I. (2020). COVID-19 crisis demonstrates the urgent need for urban greenspaces. Front. Ecol. Environ..

[24] Venter Z.S., Barton D.N., Gundersen V., Figari H., Nowell M. (2020). Urban nature in a time of crisis: Recreational use of green space increases during the COVID-19 outbreak in Oslo, Norway. Environ. Res. Lett..

[25] Pouso S., Borja A., Fleming L.E., Gómez-Baggethun E., White M.P., Uyarra M.C. (2020). Maintaining Contact with Blue-Green Spaces during the COVID-19 Pandemic Associated with Positive Mental Health.

[26] Olsen J., Mitchell R. (2020). Change in Use of Green and Open Space Following COVID-19 Lockdown ‘Stay at Home’ Phase and Initial Easing of Lockdown.

[27] Dreger S., Schüle S.A., Hilz L.K., Bolte G. (2019). Social inequalities in environmental noise exposure: A review of evidence in the WHO European Region. Int. J. Environ. Res. Public Health.

[28] Fairburn J., Schüle S.A., Dreger S., Karla Hilz L., Bolte G. (2019). Social Inequalities in Exposure to Ambient Air Pollution: A Systematic Review in the WHO European Region. Int. J. Environ. Res. Public Health.

[29] World Health Organization (WHO) (2019). Environmental Health Inequalities in Europe: Second Assessment Report.

[30] Bolte G., Bunge C., Hornberg C., Köckler H. (2018). Environmental justice as an approach to tackle environmental health inequalities. Bundesgesundheitsblatt Gesundheitsforsch. Gesundheitsschutz.

[31] Schüle S.A., Hilz L.K., Dreger S., Bolte G. (2019). Social Inequalities in Environmental Resources of Green and Blue Spaces: A Review of Evidence in the WHO European Region. Int. J. Environ. Res. Public Health.

[32] Wüstemann H., Kalisch D., Kolbe J. (2017). Access to urban green space and environmental inequalities in Germany. Landsc. Urban Plan..

[33] Nesbitt L., Meitner M.J., Girling C., Sheppard S.R.J., Lu Y. (2019). Who has access to urban vegetation? A spatial analysis of distributional green equity in 10 US cities. Landsc. Urban Plan..

[34] Rigolon A., Browning M.H.E.M., Lee K., Shin S. (2018). Access to Urban Green Space in Cities of the Global South: A Systematic Literature Review. Urban Sci..

[35] Bunge C., Rehling J. (2020). Umweltgerechtigkeit in Städten: Empirische Befunde und Strategien für mehr gesundheitliche Chancengleichheit. IzR.

[36] Schüle S.A., Gabriel K.M., Bolte G. (2017). Relationship between neighbourhood socioeconomic position and neighbourhood public green space availability: An environmental inequality analysis in a large German city applying generalized linear models. Int. J. Hyg. Environ. Health.

[37] Markevych I., Maier W., Fuertes E., Lehmann I., von Berg A., Bauer C.-P., Koletzko S., Berdel D., Sugiri D., Standl M. (2017). Neighbourhood greenness and income of occupants in four German areas: GINIplus and LISAplus. Urban For. Urban Green..

[38] Shrestha R., Flacke J., Martinez J., Van Maarseveen M. (2016). Environmental health related socio-spatial inequalities: Identifying “hotspots” of environmental burdens and social vulnerability. Int. J. Environ. Res. Public Health.

[39] Schade M. (2014). Umwelt, Soziale Lage und Gesundheit bei Kindern in Frankfurt am Main, Frankfurt am Main. Master’s Dissertation.

[40] Schulz C., Kolossa-Gehring M., Gies A. (2017). German Environmental Survey for Children and Adolescents 2014-2017 (GerES V)—The environmental module of KiGGS Wave 2. J. Health Monit..

[41] Mauz E., Gößwald A., Kamtsiuris P., Hoffmann R., Lange M., von Schenck U., Allen J., Butschalowsky H., Frank L., Hölling H. (2017). New data for action. Data collection for KiGGS Wave 2 has been completed. J. Health Monit..

[42] Bundesinstitut für Bau-, Stadt- und Raumforschung (BBSR) (2018). Handlungsziele für Stadtgrün und deren empirische Evidenz. Indikatoren, Kenn- und Orientierungswerte.

[43] Poelman H. (2016). A Walk to the Park? Assessing Access to Green Areas in Europe’s Cities.

[44] Lampert T., Hoebel J., Kuntz B., Müters S., Kroll L.E. (2018). Socioeconomic status and subjective social status measurement in KiGGS Wave 2. J. Health Monit..

[45] Bundesinstitut für Bau-, Stadt- und Raumforschung (BBSR) (2017). Gemeinsame Evaluierung der Programme Stadtumbau Ost und Stadtumbau West.

[46] Dill V., Jirjahn U., Tsertsvadze G. (2015). Residential Segregation and Immigrants’ Satisfaction with the Neighborhood in Germany. Soc. Sci. Q..

[47] Schunck R., Reiss K., Razum O. (2015). Pathways between perceived discrimination and health among immigrants: Evidence from a large national panel survey in Germany. Ethn. Health.

[48] Frank L., Yesil-Jürgens R., Born S., Hoffmann R., Santos-Hövener C., Lampert T. (2018). Maßnahmen zur verbesserten Einbindung und Beteiligung von Kindern und Jugendlichen mit Migrationshintergrund in KiGGS Welle 2. J. Health Monit..

[49] Lange M., Hoffmann R., Mauz E., Houben R., Gößwald A., Rosario A.S., Kurth B.-M. (2018). Längsschnitterhebung von KiGGS Welle 2—Erhebungsdesign und Fallzahlentwicklung der KiGGS-Kohorte. J. Health Monit..

[50] IBM Corp (2017). IBM SPSS Statistics for Windows.

[51] Taylor L., Hochuli D.F. (2017). Defining greenspace: Multiple uses across multiple disciplines. Landsc. Urban Plan..

[52] Grellier J., White M.P., Albin M., Bell S., Elliott L.R., Gascón M., Gualdi S., Mancini L., Nieuwenhuijsen M.J., Sarigiannis D.A. (2017). BlueHealth: A study programme protocol for mapping and quantifying the potential benefits to public health and well-being from Europe’s blue spaces. BMJ Open.

[53] Krekel C., Kolbe J., Wüstemann H. (2016). The greener, the happier? The effect of urban land use on residential well-being. Ecol. Econ..

[54] Coolen H., Meesters J. (2012). Private and public green spaces: Meaningful but different settings. J. Hous. Built. Environ..

